# Dr. A. P. Waid

**Published:** 1904-01

**Authors:** 


Dr. A. P. Waid, of Buffalo, a graduate of the medical department, U. of B., 1874, died at his home, December 21, 1903, aged 70 years. The remains were taken to Spartansburg, Pa., for burial.

